# The effects of synbiotic supplementation on hormonal status, biomarkers of inflammation and oxidative stress in subjects with polycystic ovary syndrome: a randomized, double-blind, placebo-controlled trial

**DOI:** 10.1186/s12902-018-0248-0

**Published:** 2018-04-12

**Authors:** Khadijeh Nasri, Mehri Jamilian, Elham Rahmani, Fereshteh Bahmani, Maryam Tajabadi-Ebrahimi, Zatollah Asemi

**Affiliations:** 1 Endocrinology and Metabolism Research Center, Arak University of Medical Sciences, Arak, Iran; 2 Research Center for Biochemistry and Nutrition in Metabolic Diseases, Kashan University of Medical Sciences, Kashan, IR Iran; 3 Faculty member of Science department, Science Faculty, Islamic Azad University, Tehran Central Branch, Tehran, Iran

**Keywords:** Synbiotic, Hormonal status, Inflammation, Oxidative stress, Polycystic ovary syndrome

## Abstract

**Background:**

To our knowledge, no reports are available indicating the effects of synbiotic supplementation on hormonal status, biomarkers of inflammation and oxidative stress in subjects with polycystic ovary syndrome (PCOS). This research was done to assess the effects of synbiotic supplementation on hormonal status, biomarkers of inflammation and oxidative stress in subjects with PCOS.

**Methods:**

This randomized double-blind, placebo-controlled trial was conducted on 60 subjects diagnosed with PCOS according to the Rotterdam criteria. Subjects were randomly assigned into two groups to take either synbiotic (*n* = 30) or placebo (*n* = 30) for 12 weeks. Endocrine, inflammation and oxidative stress biomarkers were quantified at baseline and after the 12-week intervention.

**Results:**

After the 12-week intervention, compared with the placebo, synbiotic supplementation significantly increased serum sex hormone-binding globulin (SHBG) (changes from baseline in synbiotic group: + 19.8 ± 47.3 vs. in placebo group: + 0.5 ± 5.4 nmol/L, *p* = 0.01), plasma nitric oxide (NO) (changes from baseline in synbiotic group: + 5.5 ± 4.8 vs. in placebo group: + 0.3 ± 9.1 μmol/L, *p* = 0.006), and decreased modified Ferriman Gallwey (mF-G) scores (changes from baseline in synbiotic group: − 1.3 ± 2.5 vs. in placebo group: − 0.1 ± 0.5, *p* = 0.01) and serum high-sensitivity C-reactive protein (hs-CRP) (changes from baseline in synbiotic group: − 950.0 ± 2246.6 vs. in placebo group: + 335.3 ± 2466.9 ng/mL, *p* = 0.02). We did not observe any significant effect of synbiotic supplementation on other hormonal status and biomarkers of oxidative stress.

**Conclusions:**

Overall, synbiotic supplementation for 12 weeks in PCOS women had beneficial effects on SHBG, mFG scores, hs-CRP and NO levels, but did not affect other hormonal status and biomarkers of oxidative stress.

**Trial registration:**

This study was retrospectively registered in the Iranian website (www.irct.ir) for registration of clinical trials (IRCT201509115623N53), on 2015–09-27.

## Background

Polycystic ovary syndrome (PCOS) is a common gynecological endocrine disorder related to irregular menstrual cycles and androgen excess affecting 6–12% of premenopausal women [1]. It was reported that several pro-inflammatory factors and mediators increase in subjects with PCOS, including C-reactive protein (CRP), leukocytes, cytokines, and reactive oxygen species [2]. Inflammation and oxidative stress are associated with obesity, type 2 diabetes mellitus (T2DM), hyperandrogenemia, insulin resistance as well as an increased risk of cardiovascular disease (CVD) [3].

Nowadays, there is a growing interest to use synbiotics and probiotics in diseases related to metabolic syndrome [4]. The basis of this interest derives mostly from the results of nutritional intervention studies suggest that synbiotics intake have beneficial effects on metabolic profiles, biomarkers of inflammation and oxidative stress among patients with gestational diabetes (GDM) [5], T2DM [6] and cancer [7]. In addition, gut microbiota may participate in the whole-body metabolism by affecting energy balance, insulin metabolism and inflammation related to metabolic disorders [8]. We have previously shown that consumption of the synbiotic bread for 8 weeks among participants with T2DM had beneficial effects on plasma nitric oxide (NO) and malondialdehyde (MDA) concentrations, but did not influence plasma total antioxidant capacity (TAC) and glutathione (GSH) values [9]. In another study by Ipar et al. [10], it was seen that synbiotic supplementation for 30 days in obese children had beneficial effects on lipid fractions and total oxidative stress. However, multi-species probiotics supplementation (10^10^ CFU/day) for 14 weeks did not affect biomarkers of inflammation and oxidative stress among trained men [11].

Synbiotics and probiotics may affect metabolic parameters through the effect on the production of short chain fatty acid (SCFA), decreased gene expression of inflammatory factors [12], and increased synthesis of GSH, apoptosis induction and up-regulation of oxidative pentose pathway activity [13]. To our knowledge, no reports are available indicating the effects of synbiotic supplementation on hormonal, inflammatory and oxidative parameters in subjects with PCOS. The objective of this study was to evaluate the effects of synbiotic supplementation on hormonal, inflammatory and oxidative parameters in these patients.

## Methods

### Trial design and participants

This randomized, double-blinded, placebo-controlled clinical trial, registered in the Iranian clinical trials website at: (http://www.irct.ir: IRCT201509115623N53). This study was conducted among 60 women with PCOS diagnosed according to the Rotterdam criteria [14, 15], aged 18–40 years who referred to the Kossar Clinic in Arak, Iran, from April to June 2016. Main exclusion criteria were: smokers, taking probiotic and/or synbiotic supplements, pregnant women, endocrine diseases including thyroid, diabetes and/or impaired glucose tolerance as well as gastrointestinal problems in the study.

### Ethics approval and consent to participate

The study was followed the Declaration of Helsinki guideline and was approved by the ethics committee of the Arak University of Medical Sciences (AUMS), Arak, Iran. Informed consent was taken from all subjects.

### Study protocol

At first, women were randomly allocated to receive either synbiotic supplements or placebo (*n* = 30 each group) for 12 weeks. Duration of the treatment was selected based on observed beneficial effects of probiotic supplementation on metabolic profiles in women with PCOS [16]. Randomization was done using computer-generated random numbers by a trained staff at the gynecology clinic. Randomization and allocation were concealed to the researchers and participants until the final analyses were completed. Synbiotic supplements were containing *Lactobacillus acidophilus*, *Lactobacillus casei* and *Bifidobacterium bifidum* (2 × 10^9^ CFU/g each) plus 0.8 g inulin. Synbiotic supplements and the placebo were manufactured by Tak Gen Zist Pharmaceutical Company (Tehran, Iran) and Barij Essence Pharmaceutical Company (Kashan, Iran), respectively. The compliance rate during the intervention was monitored by a brief daily cell phone reminder to take the supplement and asking the subjects to return the supplement containers. All participants completed a 3-days food record and physical activity records as metabolic equivalents (METs) prior to intervention, at weeks 3, 6, 9 and 12 of the treatment. Daily macro- and micro-nutrient intakes were calculated by analyzing food data using nutritionist IV software (First Databank, San Bruno, CA) [17].

### Anthropometric parameters

Anthropometric measurements were determined in a fasting status using a standard scale (Seca, Hamburg, Germany) at baseline and after the 12-week treatment. Body mass index (BMI) was calculated as weight in kg divided by height in meters squared.

### Clinical assessments

Clinical parameters included determinations of hirsutism using a mFG scoring system [18].

### Biochemical evaluation

At pre- and post-treatment, 10 mL blood were collected from each subject at Arak reference laboratory. Hormonal profiles were determined using an Elisa kits (DiaMetra, Milano, Italy) with inter- and intra-assay coefficient variances (CVs) lower than 7%. Free androgen index (FAI) was calculated based on suggested formulas. High sensitivity C-reactive protein (hs-CRP) and insulin values were assessed by ELISA kits (LDN, Nordhorn, Germany) and (Monobind, California, USA), respectively. The plasma NO [19], TAC [20], GSH [21] and MDA levels [22] were determined by the spectrophotometric method with inter- and intra-assay CVs less than 5%. To determine fasting plasma glucose, we used Pars Azmun kit, Tehran, Iran. The homeostatic model of assessment for insulin resistance (HOMA-IR) was determined according to suggested formulas [23].

### Sample size

We used a randomized clinical trial sample size formula with type one (*α*) and type two errors (*β*) to be 0.05 and the power of 80% to calculate sample size. Based on a previous study [24], we used a standard deviation (SD) of 283.7 ng/mL and a difference in mean (d) of 230.0 ng/mL, considering hs-CRP levels as the key variable. According to the calculation 25 women should be enrolled in each group. Assuming a dropout of 5 subjects per group, the final sample size was considered to be 30 per treatment group.

### Statistical methods

The Kolmogorov-Smirnov test was performed to determine the normality of data. Outcome log-transformation was used if model residual has non-normal distribution (hs-CRP, MDA, SHBG and FAI). To detect differences in anthropometric parameters as well as in macro- and micro-nutrient intakes between the two groups, we applied independent *t*-test. To assess the effects of synbiotic supplementation on metabolic parameters, we used one-way repeated measures analysis of variance. Adjustment for changes in baseline values of biochemical parameters, age and baseline BMI was performed by analysis of covariance (ANCOVA). *P*-values < 0.05 were considered statistically significant. All statistical analyses were done using the Statistical Package for Social Science version 18 (SPSS Inc., Chicago, Illinois, USA).

## Results

**Fig. 1:**
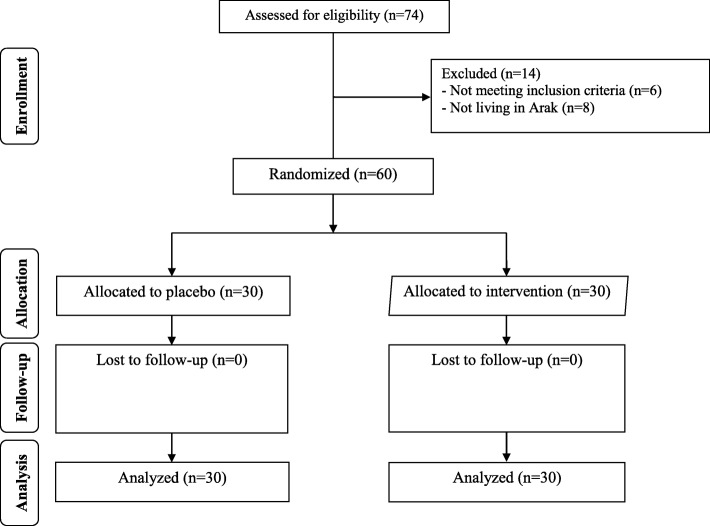
Summary of patient flow diagram

In this study, all 60 subjects [synbiotic and placebo (*n* = 30 each group)] completed the trial (Fig. 1). The compliance rate in this study was high; more than 90% of capsules were taken during the course of the trial in both groups. No side effects were reported following the intake of synbiotic supplements in patients with PCOS.

Mean age, height, and weight, BMI and METs at baseline and end-of-trial were not statistically different between the two groups (Table 1).

**Table 1:** General characteristics of study participants

	Placebo group (*n* = 30)	Synbiotic group (*n* = 30)	*p*^a^
Age (y)	25.9 ± 5.2	25.7 ± 5.5	0.90
Height (cm)	163.3 ± 6.6	161.4 ± 5.8	0.25
Weight at study baseline (kg)	72.4 ± 14.1	71.4 ± 11.6	0.79
Weight at end-of-trial (kg)	71.9 ± 14.4	71.2 ± 11.4	0.83
Weight change (kg)	−0.4 ± 1.0	− 0.3 ± 1.2	0.53
BMI at study baseline (kg/m^2^)	27.2 ± 5.3	27.4 ± 4.0	0.84
BMI at end-of-trial (kg/m^2^)	27.0 ± 5.4	27.3 ± 3.9	0.80
BMI change (kg/m^2^)	−0.2 ± 0.3	− 0.1 ± 0.4	0.49
MET-h/day at study baseline	27.5 ± 2.0	27.7 ± 2.1	0.60
MET-h/day at end-of-trial	27.6 ± 2.2	27.8 ± 2.3	0.69
MET-h/day change	0.1 ± 0.6	0.04 ± 1.0	0.83

Data are means± SDs

^a^Obtained from independent *t* test. METs, metabolic equivalents

No significant difference in mean dietary macro- and micro-nutrient intakes between the two groups was seen (Data not shown).

Compared with the placebo, synbiotic supplementation significantly increased serum sex hormone-binding globulin (SHBG) (changes from baseline in synbiotic group: + 19.8 ± 47.3 vs. in placebo group: + 0.5 ± 5.4 nmol/L, *p* = 0.01), plasma NO (changes from baseline in synbiotic group: + 5.5 ± 4.8 vs. in placebo group: + 0.3 ± 9.1 μmol/L, *p* = 0.006), and decreased mF-G scores (changes from baseline in synbiotic group: − 1.3 ± 2.5 vs. in placebo group: − 0.1 ± 0.5, *p* = 0.01), FAI (changes from baseline in synbiotic group: − 0.12 ± 0.29 vs. in placebo group: − 0.01 ± 0.08, *p* = 0.01) and serum hs-CRP (changes from baseline in synbiotic group: − 950.0 ± 2246.6 vs. in placebo group: + 335.3 ± 2466.9 ng/mL, *p* = 0.02) (Table 2). In addition, compared with the placebo, synbiotic supplementation resulted in a significant reduction in serum insulin levels (changes from baseline in synbiotic group: − 1.6 ± 2.9 vs. in placebo group: + 0.4 ± 2.3 μIU/mL, *p* = 0.003), HOMA-IR (changes from baseline in synbiotic group: − 0.4 ± 0.7 vs. in placebo group: + 0.1 ± 0.5, *p* = 0.003). A trend toward a greater decrease in total testosterone (changes from baseline in synbiotic group: − 0.4 vs. in placebo group: − 0.1 ng/mL, *p* = 0.09) and plasma MDA concentrations (changes from baseline in synbiotic group: − 0.2 ± 0.1 vs. in placebo group: + 0.5 ± 1.4 μmol/L, *p* = 0.05) was observed in synbiotic group compared with placebo group. We did not observe any significant effect of synbiotic supplementation on other hormonal status and biomarkers of oxidative stress.

**Table 2:** Hormonal status, biomarkers of inflammation and oxidative stress at baseline and after the 12-week intervention in subjects with polycystic ovary syndrome

	Placebo group (*n* = 30)			Synbiotic group (*n* = 30)			*p*^a^
	Baseline	End-of-trial	Change	Baseline	End-of-trial	Change	
Total testosterone (ng/mL)	2.4 ± 1.2	2.3 ± 1.0	−0.1 ± 0.5	2.8 ± 1.3	2.4 ± 0.9	−0.4 ± 0.9	0.09
SHBG (nmol/L)	38.3 ± 17.3	38.8 ± 17.6	0.5 ± 5.4	37.3 ± 13.1	57.1 ± 48.6	19.8 ± 47.3	0.01
FAI	0.27 ± 0.21	0.25 ± 0.16	−0.01 ± 0.08	0.33 ± 0.36	0.21 ± 0.14	−0.12 ± 0.29	0.01
mF-G scores	15.1 ± 3.8	15.0 ± 3.7	−0.1 ± 0.5	15.3 ± 5.6	14.0 ± 4.9	−1.3 ± 2.5	0.01
DHEAS (μg/mL)	2.6 ± 1.3	2.5 ± 1.1	−0.1 ± 0.4	2.6 ± 1.5	2.2 ± 0.8	−0.4 ± 1.1	0.40
hs-CRP (ng/mL)	2990.7 ± 2510.7	3326.0 ± 2791.1	335.3 ± 2466.9	2920.0 ± 2251.2	1970.0 ± 1442.0	−950.0 ± 2246.6	0.02
NO (μmol/L)	40.5 ± 8.7	40.8 ± 9.3	0.3 ± 9.1	39.0 ± 3.1	44.5 ± 5.0	5.5 ± 4.8	0.006
TAC (mmol/L)	868.7 ± 158.4	877.9 ± 149.9	9.2 ± 119.3	773.1 ± 38.7	818.2 ± 57.5	45.1 ± 51.8	0.13
GSH (μmol/L)	494.2 ± 85.5	521.5 ± 117.2	27.3 ± 117.8	498.9 ± 56.8	523.5 ± 53.4	24.7 ± 58.7	0.91
MDA (μmol/L)	2.2 ± 0.7	2.7 ± 1.2	0.5 ± 1.4	2.3 ± 0.4	2.1 ± 0.4	−0.2 ± 0.1	0.05

All values are means± SDs

^a^*P* values represent the time × group interaction (computed by analysis of the one-way repeated measures ANOVA)

*DHEAS* dehydroepiandrosterone sulfate, *FAI* free androgen index, *GSH* total glutathione, *hs-CRP* high-sensitivity C-reactive protein, *mF-G* modified Ferriman Gallwey, *MDA* malondialdehyde, *NO* nitric oxide, *SHBG* sex hormone-binding globulin, *TAC* total antioxidant capacity

Baseline levels of plasma TAC (*p* = 0.002) were significantly different between the two groups. Therefore, we controlled the analyses for the baseline levels, age and baseline BMI. When we adjusted the analyses for baseline values of biochemical variables, age and baseline BMI, significant changes in FAI (*p* = 0.04) were observed, but other findings did not alter (Table 3).

**Table 3:** Adjusted changes in metabolic profile of the patients with polycystic ovary syndrome

	Placebo group (*n* = 30)	Synbiotic group (*n* = 30)	*p*^a^
Total testosterone (ng/mL)	− 0.2 ± 0.1	− 0.3 ± 0.1	0.26
SHBG (nmol/L)	0.7 ± 6.1	19.5 ± 6.1	0.03
FAI	−0.04 ± 0.02	−0.10 ± 0.02	0.04
mF-G scores	−0.1 ± 0.3	−1.3 ± 0.3	0.007
DHEAS (μg/mL)	−0.1 ± 0.1	−0.3 ± 0.1	0.18
hs-CRP (ng/mL)	375.6 ± 339.8	−990.2 ± 339.8	0.006
NO (μmol/L)	0.6 ± 1.2	5.2 ± 1.2	0.009
TAC (mmol/L)	23.8 ± 16.3	30.5 ± 16.3	0.78
GSH (μmol/L)	26.3 ± 15.8	25.7 ± 15.8	0.98
MDA (μmol/L)	0.4 ± 0.2	−0.1 ± 0.2	0.02

All values are means± SEs. Values are adjusted for baseline values, age and BMI at baseline

^a^Obtained from ANCOVA

*DHEAS* dehydroepiandrosterone sulfate, *FAI* free androgen index, *GSH* total glutathione, *hs-CRP* high-sensitivity C-reactive protein, *mF-G* modified Ferriman Gallwey, *MDA* malondialdehyde, *NO* nitric oxide, *SHBG* sex hormone-binding globulin, *TAC* total antioxidant capacity

## Discussion

In this research, which to our knowledge is the first of its kind, we assessed the effects of synbiotic supplementation on hormonal, inflammatory and oxidative parameters among subjects with PCOS. We shown that taking synbiotic supplements for 12 weeks among PCOS subjects had beneficial effects on SHBG, mFG scores, FAI, serum insulin, HOMA-IR, serum hs-CRP and plasma NO levels, but did not affect other hormonal, inflammatory and oxidative parameters. However, observed reduction at mFG scores after 12 weeks was statistically significant, it was clinically low. Long-term interventions and higher dosage of probiotic and inulin might result in greater changes in mFG scores.

Subjects with PCOS are susceptible to several metabolic complications including insulin resistance and inflammation [25, 26]. We found that synbiotic administration for 12 weeks among PCOS subjects led to a significant increase in serum SHBG values and FAI and a significant decrease in mFG scores, serum insulin levels and HOMA-IR, but did not affect hormonal profiles compared with the placebo. However, to our knowledge, no reports are available indicating the effects of synbiotic supplementation on hormonal status, biomarkers of inflammation and oxidative stress in subjects with PCOS; some studies have evaluated the effects of synbiotic supplementation on markers of insulin metabolism among subjects without PCOS. We have previously shown that taking synbiotic supplements for 6 weeks among subjects with GDM had beneficial effects on markers of insulin metabolism [5]. Shoaei et al. [27] also indicated that probiotic supplementation for 12 weeks to women with PCOS significantly decreased fasting glucose and insulin concentrations. In another study conducted by Eslamparast et al. [28], it was seen that levels of fasting glucose and insulin resistance were improved significantly in the synbiotic group among subjects with metabolic syndrome after 28 weeks. In addition, the intake of synbiotic containing *Lactobacillus acidophilus*, *Bifidobacterium bifidum* and fructo-oligosaccharides in elderly people with T2DM resulted in a significant reduction in fasting glycemia [29]. Hyperinsulinemia and insulin resistance in women with PCOS directly stimulate ovarian steroidogenesis by acting on thecal cell proliferation and increasing secretion of androgens mediated by luteinizing hormone (LH), increased gene expression of cytochrome P450 and insulin-like growth factor 1 receptor [30]. In addition, androgens may regulate follicular atresia [31]. It was also reported that increased testosterone levels increase somatic cell atresia in rat ovaries [32]. Furthermore, hyperandrogenemia can induce inflammation in women with PCOS [33]. Therefore, synbiotic intake due to its useful effects on insulin resistance may be useful to control clinical and metabolic symptoms. Synbiotic intake might improve SHBG and mFG scores through improved insulin sensitivity, the modification of gut flora, the elevation of faecal pH [34] and the reduction of pro-inflammatory cytokine production [35].

Our previous study among subjects with T2DM has demonstrated that consumption of a synbiotic food for 6 weeks had significant effects on serum hs-CRP concentrations [24]. In addition, supplementation with a synbiotic among adults with nonalcoholic fatty liver disease over 28 weeks inhibited inflammatory markers [36]. Consumption of the synbiotic bread for 2 months in people with T2DM significantly increased plasma levels of NO and decreased MDA, but unchanged TAC, GSH, catalase concentrations [9]. These findings were similar in pregnant women [37] and patients with rheumatoid arthritis [38]. Furthermore, soy milk containing probiotic for 48 h increased NO production in human endothelial cells [39]. A significant decline in MDA values was also evidenced after the intake of probiotic in rabbits for 30 days [40]. However, synbiotic supplementation for 6 weeks did not influence CRP values [41]. In addition, NO status did not affect by probiotic in herpes simplex virus type 1 [42]. Supplementation with probiotic supplements for 7 days did not decrease MDA values [43]. Elevated inflammatory markers in subjects with PCOS would result in increased risk of atherosclerosis, diabetes and infertility [44]. In addition, oxidative stress is correlated with obesity and hyperandrogenism [45]. Increased oxidative stress could also induce directly genetic variation by DNA damage, and epigenetic change including elevated DNA methylation levels, which both play important roles in the pathogenesis of cancer [46, 47]. Up-regulation of IL-18 by SCFA products [48] and elevated production of methylketones in gut by synbiotic [49] might decrease inflammatory markers. Decreased hydroperoxides by synbiotic intake may elevate NO levels [50, 51]. Moreover, synbiotic intake may reduce MDA because its impact on decreased lipid parameters [52] and inhibiting lipid peroxidation reactions [53, 54].

Limitations of our study include the absent of testing for a dose-response relationship between synbiotic intake and occurred changes in the metabolic profiles. Furthermore, we did not determine the effects of synbiotic on other metabolic parameters. However, duration of the treatment was too short to determine the effects of synbiotic on hormonal parameters and mFG scores; we believe that future studies with cross-over design and longer duration of the intervention are required to prove our findings. Furthermore, the high standard deviations (SDs) of dependent parameters in some cases might be due to the small number of participants in the study.

## Conclusions

Overall, synbiotic supplementation for 12 weeks in PCOS women had beneficial effects on SHBG, mFG scores, FAI, hs-CRP and NO levels, but did not affect other hormonal status and biomarkers of oxidative stress.

## Abbreviations

CVD: Cardiovascular disease
CVs: Coefficient variances
DHEAS: Dehydroepiandrosterone sulfate
FAI: Free androgen index
GDM: Gestational diabetes
GSH: Total glutathione
hs-CRP: High-sensitivity C-reactive protein
MDA: Malondialdehyde
mF-G: Modified Ferriman Gallwey
NO: Nitric oxide
PCOS: Polycystic ovary syndrome
SCFA: Short chain fatty acid
SHBG: Sex hormone-binding globulin
T2DM: Type 2 diabetes mellitus
TAC: Total antioxidant capacity
